# Manipulating the Sensitivity of Signal-Induced Repression: Quantification and Consequences of Altered Brinker Gradients

**DOI:** 10.1371/journal.pone.0071224

**Published:** 2013-08-08

**Authors:** Lucia Gafner, Sascha Dalessi, Eliane Escher, George Pyrowolakis, Sven Bergmann, Konrad Basler

**Affiliations:** 1 Institute of Molecular Life Sciences, University of Zurich, Zurich, Switzerland; 2 Department of Medical Genetics, University of Lausanne, Lausanne, Switzerland and SIB Swiss Institute of Bioinformatics, Lausanne, Switzerland; 3 Department of Developmental Biology and BIOSS Centre for Biological Signalling Studies, University of Freiburg, Freiburg, Germany; Simon Fraser University, Canada

## Abstract

Traditionally, the analysis of gene regulatory regions suffered from the caveat that it was restricted to artificial contexts (e.g. reporter constructs of limited size). With the advent of the BAC recombineering technique, genomic constructs can now be generated to test regulatory elements in their endogenous environment. The expression of the transcriptional repressor *brinker (brk)* is negatively regulated by Dpp signaling. Repression is mediated by small sequence motifs, the *silencer elements (SEs)*, that are present in multiple copies in the regulatory region of *brk*. In this work, we manipulated the *SEs* in the *brk* locus. We precisely quantified the effects of the individual *SEs* on the Brk gradient in the wing disc by employing a 1D data extraction method, followed by the quantification of the data with reference to an internal control. We found that mutating the *SEs* results in an expansion of the *brk* expression domain. However, even after mutating all predicted *SEs*, repression could still be observed in regions of maximal Dpp levels. Thus, our data point to the presence of additional, low affinity binding sites in the *brk* locus.

## Introduction

The *Drosophila* wing imaginal disc is routinely used as a model to study growth and patterning. In the first larval instar, it consists of about 40 cells. At metamorphosis around four days later, the cell number has increased up to 100 000 [1]. Growth and patterning of the wing imaginal disc are regulated by gradients of morphogens. Key examples are *wingless (wg)*, which is expressed along the dorso-ventral (D/V) and *decapentaplegic (dpp)*, which is expressed along the anterior-posterior (A/P) compartment boundary.

From its source Dpp spreads both into the anterior and the posterior compartment, forming a concentration gradient. Binding of the ligand Dpp to its receptors, Thick veins and Punt, triggers phosphorylation of the *Drosophila* receptor-regulated R-Smad protein Mad. Two phosphorylated Mad (pMad) subunits form a complex with the co-Smad Medea [2]. Upon migration to the nucleus this complex directly activates the transcription of Dpp target genes. For most target genes this activating branch of the Dpp pathway plays only a minor role. Instead, the main mechanism of Dpp target gene activation is the Dpp signaling mediated downregulation of their default repressor, *brinker (brk)* [3]–[5]. Some target genes seem to be exclusively regulated via Brk (e.g. *optomotorblind, omb; bifid*) while the expression of others seems to depend on a combination of direct activation and *brk* repression (e.g. *spalt, sal; spalt major, salm*) [3], [4]. The repression of *brk* has been termed “signal-induced repression” and represents an example of an interesting but poorly understood mechanism that can also be found in other pathways (for review: [6]).

When repressing *brk*, the tripartite pMad-pMad-Med complex binds to short cis-regulatory elements, the so-called *silencer elements (SEs),* in the *brk* locus and subsequently recruits and forms a complex with the large nuclear zinc finger protein Schnurri (Shn) [7]. The *SEs* were shown to share the consensus sequence GRCGNC (N)_5_ GTCTG, where the first motif GRCGNC is bound by Mad, while Med recognizes the motif GTCTG [2], [8]. Binding of the pathway mediators to the *SEs* results in transcriptional repression of the *brk* gene.

Hence, the Dpp morphogen gradient and *brk* expression form complementary gradients in the wing imaginal disc, with high Brk levels only in lateral regions – or at the “brink”. Brk recognizes and binds the target site GGCGYY [9]–[12]. Dpp pathway target genes, such as *sal* or *omb*, are expressed in defined, nested domains in the center of the wing imaginal disc; the domains have different widths in accordance with their differential sensitivity to Brk mediated repression [3], [4], [11], [13], [14].

Clearly, in order to understand how Dpp controls growth and patterning, we need to understand how *brk* expression is regulated. Previous studies have suggested a modular nature for the *brk* locus. Various genomic fragments can reproduce the endogenous *brk* expression pattern when tested in reporter constructs. These fragments must contain both *SEs* and enhancers. Furthermore, it was reported that the enhancer sequences are located no more than 380 bp away from the corresponding *SEs* [15].

So far, it has remained elusive how the proposed combinations of *SEs* and enhancers affect *brk* expression in the context of the entire genomic *brk* locus and what would be the effect of providing only single *SEs* or few functional *SEs*, in a locus otherwise depleted of functional *SEs*. Here, we addressed this question by making use of large genomic constructs in which the expression of *brk* is monitored by the expression of fluorescent proteins. To consolidate our findings, we also established a sophisticated quantification method, with which we can detect and quantify even very subtle changes in the Brk gradient.

## Results

### Generation of a Genomic *brk* Reporter Construct

**Figure 1:**
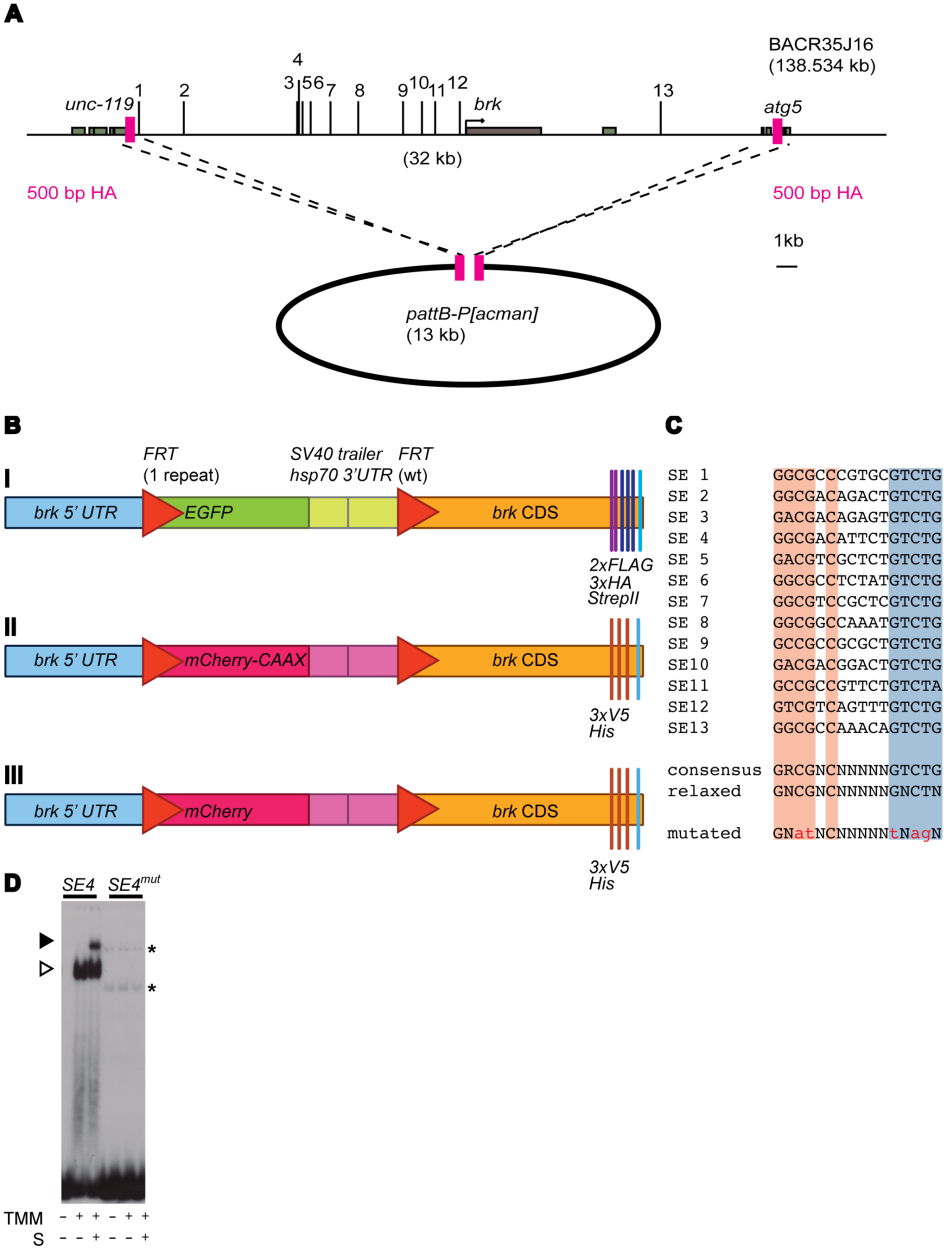
Generation of tagged and fluorescently labeled genomic *brk* constructs. (A) Homologous recombination mediated retrieval of the genomic region of interest into the integration vector *pattB-P[acman]*. Schematic overview of the *brk* locus, the distribution of the 13 putative *SEs* is indicated. The *brk* coding sequence and the up- and downstream neighboring genes (*unc-119* and *atg5*, respectively) are shown in gray, 500 bp homology arms (HA) in pink. Fragment B14 from Muller et al. contains *SEs* 3–8 [14]. *SEs* 11–2 from Yao et al. correspond to *SEs* 1–10 in this current study; *SE* 1 corresponds to *SE* 12 in this current study [15]. (B) Schematic overview of the *EGFP* and *mCherry* flip out cassettes as well as the C-terminal tags. (I) The *EGFP* flip out cassette and C-terminal FLAG-, HA-tags and strep-tagII that were introduced in the 10 constructs that were generated in this work, featuring from zero to 13 functional *SEs*. (II) A membrane-targeted *mCherry* in a flip out cassette and C-terminal V5- and His-tags were used in the wild type construct that served as an internal control for the quantification. (III) Construct similar to the one above, except that the mCherry in this construct lacks the CAAX motif and is hence no longer recruited to the cell membrane (see Text S1). (C) Sequence summary of the 13 putative *SEs*. The Mad and Med binding motifs are shown in red and blue, respectively. A spacer of five random nucleotides and the T at position 15 allow Shn binding. Shown is the consensus sequence as previously described (“consensus” according to [2], [8]). For this project, we allowed the consensus to become more degenerate, or “relaxed”, allowing more mismatches compared to the original consensus sequence. The five point mutations that were introduced into each *SE* are shown in lower case (“mutated”). (D) EMSA with *SE4* as a representative example to examine whether the introduced mutations prevent complex formation. Results shown for both wild type *SE4* and *SE4mut*. Each labeled DNA was incubated with extracts of mock transfected cells (first lane) or extracts of cells transfected with TkvQD, Mad and Medea (TMM) in the absence (second lane) or presence (third lane) of ShnCT (S). Open arrow: Mad-Med shift, closed arrow: Mad-Med-ShnCT super shift. Nonspecific binding events are indicated by asterisks.

To express *brk* under the control of its endogenous regulatory sequences, 32 kb of the *brk* locus were included in the final construct (Fig. 1A). A *FRT* flanked *EGFP* (*enhanced GFP*) stop cassette was introduced into the *brk* 5′ UTR. To be able to analyze Brk protein levels upon removal of the *EGFP* stop cassette, we also tagged Brk at its C-terminus (Fig. 1B). The resulting construct is denoted as *allSEwt>EGFP>brk-FLAG-HA-strepII*. Expression of the *EGFP* is under the control of the endogenous *brk* regulatory region and thus serves as a transcriptional *brk* reporter. The sequence was transferred into the integration vector *pattB-P[acman]* (Fig. 1A; [16]) and transgenic flies were generated by means of ΦC31 integrase mediated site-specific integration [17].

**Figure 2:**
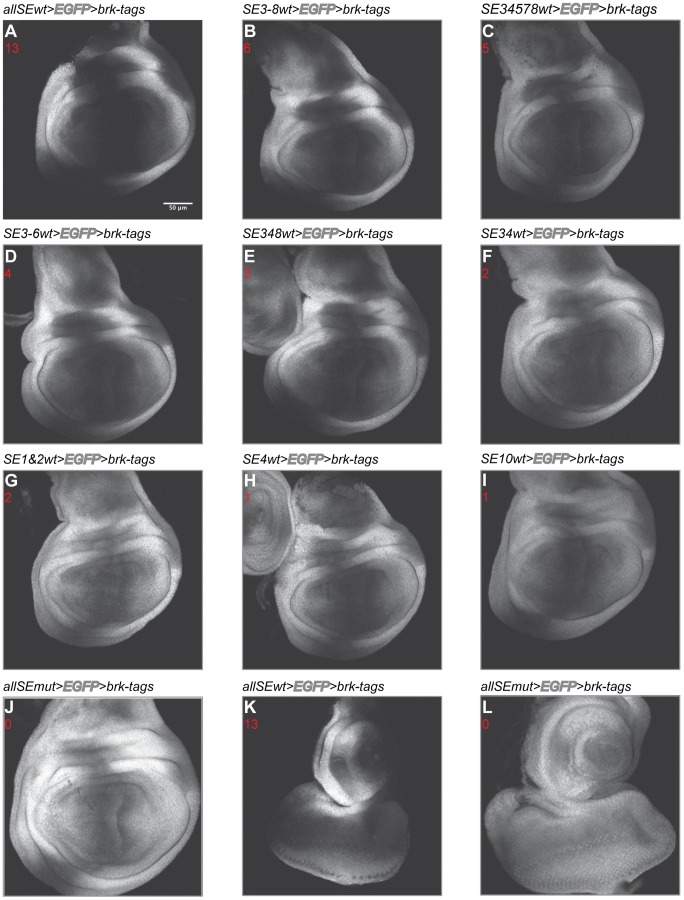
Mutating the *SEs* results in a medial expansion of the *EGFP* expression domain. *EGFP* expression patterns resulting from different subsets of functional and mutated *SEs*. The expression pattern seems to be a function of both the number and identity of the functional *SEs*. Genotypes are indicated. The number of functional *SEs* present in the constructs is given in red. (A)-(J) Expression patterns in the wing imaginal disc. (K)-(L) Expression patterns in the eye disc. Scale bar 50 µm. Pictures taken with constant confocal settings.

In wing imaginal discs dissected from larvae transgenic for this control construct *(allSEwt>EGFP>brk-FLAG-HA-strepII)*, the endogenous *brk* expression pattern was perfectly recapitulated by the *EGFP* readout (Fig. 2A). To confirm the functionality of the tagged Brk protein and to analyze the phenotypic effects of manipulating the *SEs* in the *brk* locus *in vivo*, we removed the *FRT* flanked *EGFP* stop cassette in transgenic flies. Germ line specific flip out of the *EGFP* cassette could rescue *brk* null mutant flies (*Brk^XH^*; [3]), demonstrating that the construct is fully functional. Furthermore, the rescued flies were phenotypically wild type (data not shown).

### The Number of Functional *SEs* Contained in a Construct is Reflected in the *EGFP* Expression

Thirteen potential *SEs* are predicted in the *brk* locus using a more degenerate consensus sequence than the one that was previously published: GNCGNC (N)_5_ GNCTN instead of GRCGNC (N)_5_ GTCTG (Fig. 1A, C; [2], [8]).

To determine the contribution of individual *SEs* to Dpp signaling mediated repression of *brk*, we tested a series of constructs carrying different combinations of functional *SEs*. Nonfunctional *SEs* were generated by introducing five point mutations, which completely abrogated binding of Mad, Med and Shn *in vitro* in electrophoretic mobility shift assays (Fig. 1C, D). Furthermore, we also biochemically examined Mad-Med-Shn complex formation on each of the 13 predicted *SEs* in the *brk* locus. A signal-induced Mad-Med-Shn complex formed on all the sites, again suggesting functionality, also for the more degenerate *SEs* (Fig. S1; *SEs* 9, 11 and 12). In addition, our assays showed that the 13 *SEs* of *brk* seem to vary in their affinity for the Smad complex and/or to recruit Shn once bound by Mad and Med. Thus, already our *in vitro* results suggest that the *SEs* might differ in their relative contribution to the BMP-dependent downregulation of *brk* expression.

Our experimental approach to introduce the changes into the genomic *brk* construct included a combination of site-directed mutagenesis and recombineering. Mutating all putative *SEs* (*allSEmut>EGFP>brk-FLAG-HA-strepII*) led to an *EGFP* expression domain that was clearly expanded into medial regions of the wing imaginal disc. This effect can be ascribed to the transcriptional derepression caused by the inactivation of the *SEs*. Unexpectedly, the derepression was not complete and in medial regions of the wing disc there was residual repression (Fig. 2J). The same was true for other tissues: In eye-antennal discs, mutating all *SEs* similarly resulted in a clear expansion of the *EGFP* expression domain compared to the control construct, yet complete derepression was not achieved (Fig. 2K, L). This interesting and surprising observation – with all *SEs* mutated, we had expected a uniform expression – will be discussed later.

First however, we will focus on the contributions of the different, known *SEs* to *brk* expression. To study this, constructs featuring an intermediate number of functional *SEs* (between one and six functional *SEs*) were generated (Fig. 2B–I). The observed expression patterns in the wing imaginal disc could be categorized as broader than the *allSEwt*, but narrower than the *allSEmut* expression pattern.

Therefore, it was evident that we needed to establish a method to extract quantitative data from the images to gain more biological insights.

### Quantification of the Brk Gradient Relative to a Differently Labeled Internal Control

To systematically, reproducibly and rigorously quantify the Brk gradients in the different *SE* mutant variants relative to the endogenous *brk* expression pattern in wing imaginal discs, we needed an internal control to serve as a reference.

Therefore, we generated an alternative wild type control construct, replacing the *EGFP* with *mCherry*. We chose a *mCherry* fused to the small GTPase derived CAAX motif, which targets the protein to the plasma membrane, as this version was shown to be functional in flies [18]. We also changed the tags and introduced V5- and His-tags; the resulting construct is designated as *allSEwt>mCherry-CAAX>brk-V5-His* (Fig. 1B). Our results are independent of the subcellular mCherry localization, as we also tested a cytoplasmic mCherry as a reference, with similar results (Fig. 1B and Text S1).

Wing imaginal discs from animals homozygous for the *allSEwt>mCherry-CAAX>brk-V5-His* construct on the second (internal control; landing site 22A) and the several mutant constructs on the third chromosome (86Fb) were dissected and recorded. By following a stringent protocol, we minimized variability between rounds of dissections and imaging. For the analysis of the resultant z-stacks of the third instar wing imaginal discs, we have developed a rigorous quantification strategy.

**Figure 3:**
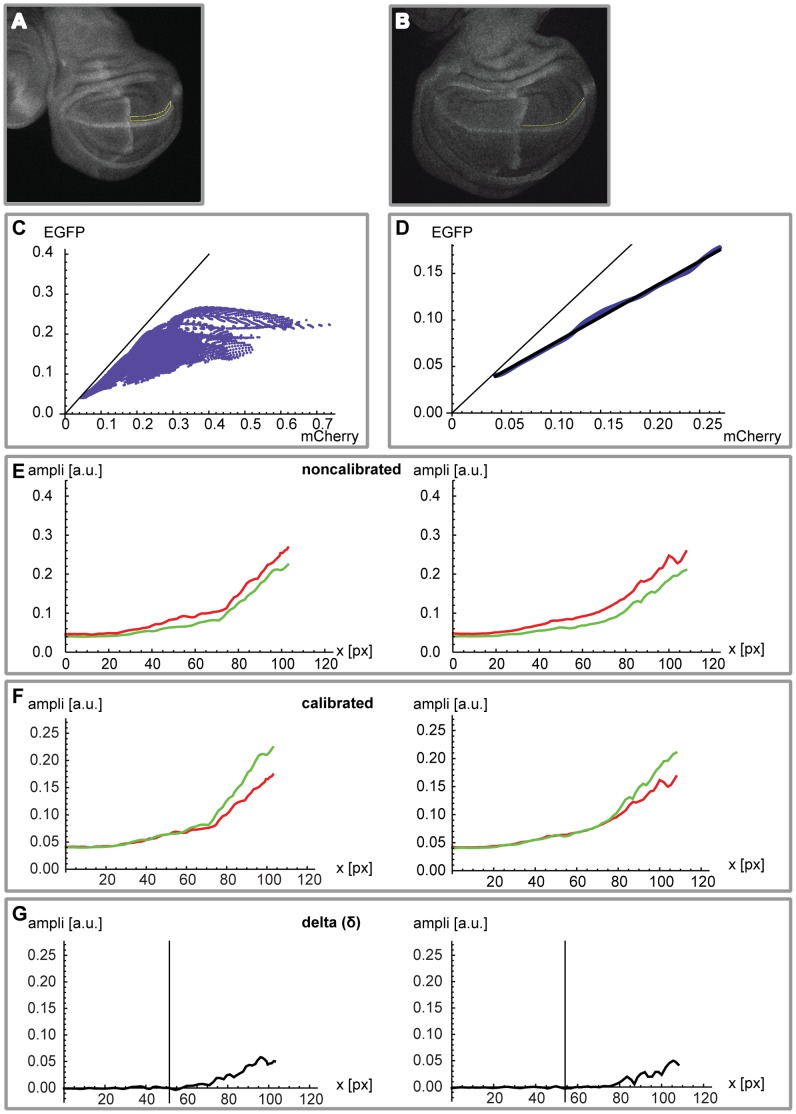
Calibration of EGFP and mCherry wild type profiles. (A) Example of a 2D mask along the *wingless* expression domain in the posterior compartment of the pouch used to extract EGFP and mCherry pixel amplitude pairs. (B) Example of a 1D ROI used for profile extraction. The line was manually drawn parallel to the *wg* expression domain, around ten pixels into the dorsal compartment. (C) All the pixel pairs (absolute fluorescence mCherry versus EGFP) collected from the 2D mask (cf. A), resulting in a cone-shaped distribution. Data for all analyzed *allSEwt>EGFP>brk-tags* discs (n = 35) was pooled prior to analysis. (D) In black, the calibration profile obtained by a linear fit of the cleaned data (cf. Text S1 for more details). (E) Two representative *allSEwt>EGFP>brk-tags* (green) versus *allSEwt>mCherry-CAAX>brk-tags* (red) profiles (absolute values, expressed in arbitrary units) for the posterior half of the pouch prior to calibration. Overall, the mCherry profiles show higher levels than the EGFP profiles. The distance (x-axis) is always expressed in pixels (1 px = 0.664 µm). (F) Same profiles as in (E), after application of the calibration to the mCherry curves (the EGFP profiles remain unchanged). The profiles become quite similar in the medial region of interest. (G) Finally, we plot the difference δ = EGFP – mCherry. As expected, values are close to zero in the medial region (medial 50% of the posterior part of the pouch; marked by vertical line).

In a first step we extract and calibrate 1D profiles (Fig. 3A–F; cf. Material and Methods and Text S1). The extraction was computed in the dorsal compartment, parallel to the D/V boundary – the expression of *ptc* was used to identify the A/P compartment boundary. The calibration procedure is necessary because of the different absolute fluorescent levels of the membrane-targeted mCherry *(allSEwt>mCherry-CAAX>brk-V5-His)* and the cytoplasmic EGFP *(allSEwt>EGFP>brk-FLAG-HA-strepII)*. In Fig. 3E we show two examples of noncalibrated profiles, and in Fig. 3F the calibration is applied to the mCherry profile. High variability was observed in peripheral regions of the pouch where the expression profiles showed maximal levels (cf. Fig. 3G, representing the absolute difference δ between the two profiles). To exclude these noisy regions from the quantification, the analysis was restricted to the medial 50% of the posterior half of the pouch (70% and 100% of the posterior half of the pouch were tested additionally; see Text S1). The aim was to optimize the signal-to-noise ratio.

**Figure 4:**
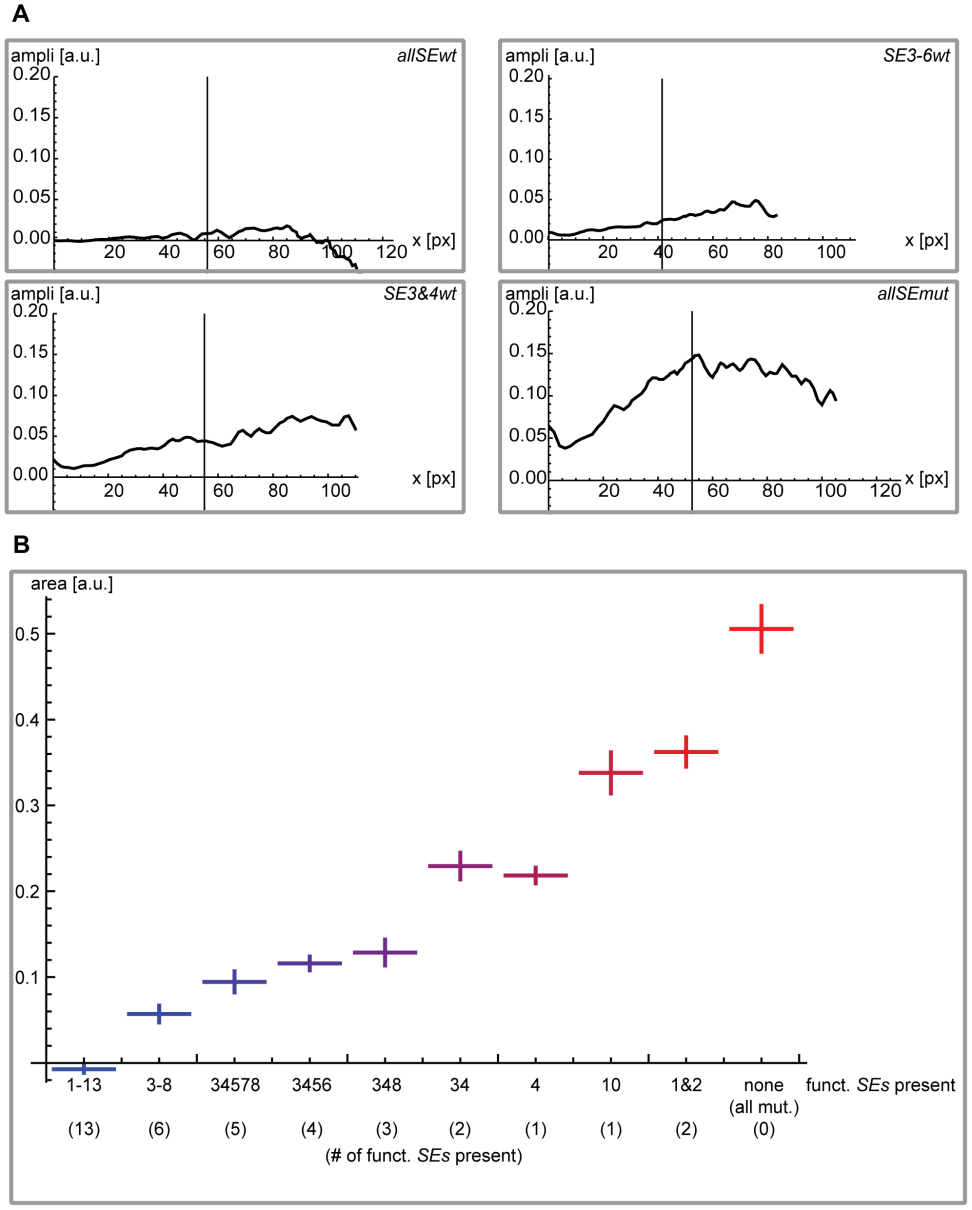
Quantification of the individual EGFP profiles for the different constructs, relative to the internal mCherry wild type control. (A) Four examples of individual wing discs carrying the indicated constructs. As in Fig. 3G, the black lines represent the difference between the EGFP and the mCherry profiles (δ = EGFP – mCherry). The vertical line again marks the medial 50% of the posterior part of the pouch. (B) Summary of the results for all the different constructs. We show, for each construct, the absolute area (medial 50% of the posterior part of the pouch) below the black δ curve (cf. A) divided by the absolute area below the red mCherry curve. Error bars represent ± two times the standard deviation for the corresponding construct. For each construct, between 6 *(SE10wt)* and 35 *(allSEwt)* individual wing discs were analyzed (*allSEwt* n = 35, *SE3-8wt* n = 14, *SE3&4&5&7&8wt* n = 12, *SE3-6wt* n = 9, *SE3&4&8wt* n = 11, *SE3&4wt* n = 7, *SE4wt* n = 8, *SE10wt* n = 6, *SE1&2wt* n = 13, *allSEmut* n = 9). Taking into account only 50% of the profile gives the best results (lowest standard deviations). Different cases (namely 70% or 100%) are presented in Text S1. The color code employed in this Figure is reused in the additional plots that can be found in Text S1.

In a second step we provide a quantitative description of the degree of derepression caused by the different combinations of mutated and functional *SEs* provided in the *brk* regulatory region for each construct and to describe every single wing disc analyzed with a single value (Fig. 3B, G and Fig. 4). We analyzed at least 6 discs for each construct and calculated the difference δ between the two profiles. In Fig. 4A we present an example of δ for different kinds of combinations of mutated *SEs* and in Fig. 4B the final quantification of the derepression (for details see Text S1).

The single *SEs* clearly differ in their respective repressive potential. The combination of functional *SE3&4* results in significantly higher repression than the combination of functional *SE1&2; SE10* causes a higher repression than *SE1&2 *but less than *SE3&4*. *SE4* stands out as a very strong *SE*, which correlates well with its high affinity for Mad, Med and Shn (see Fig. S1). The combination of functional *SE3&4* does not further enhance repression than providing *SE4* exclusively (Fig. 4B). None of the individual *SEs* reconstituted the endogenous *brk* expression pattern.

In summary, we present a new gradient quantification approach that provides us with precise and reproducible results of how Dpp signaling mediated repression via the *SEs* affects the Brk gradient. We found that providing individual or combinations of a few functional *SEs* does not suffice to restore the endogenous *brk* expression pattern. Moreover, individual *SEs* show unequal repressive properties.

### A Certain Threshold of Functional *SEs* Needs to be Crossed to Ensure Viability

The role of Brk in growth and patterning control makes it imperative that its levels are tightly controlled. The next question we asked was what degree of repression is required for viability? To this end we examined the viability of flies expressing *brk* under the control of different combinations of *SEs*. Providing only three functional *SEs (SE3&4&8)* resulted in an *EGFP* expression pattern that showed an intermediate level of derepression (Fig. 2E and 4B). We tested this construct *(SE3&4&8)* and the construct carrying one less functional *SE (SE3&4)* for their ability to rescue *brk* null mutant flies upon FLP mediated removal of the *EGFP* cassette. The construct comprising three functional *SEs (SE3&4&8)* allowed a full rescue and the resulting flies showed no phenotypic anomalies. Adult wing length, wing width and wing area were measured and were nearly identical for the wild type construct and the construct featuring wild type *SE3&4&8*. In contrast, the construct featuring two functional *SEs (SE3&4)* resulted in pupal lethality (data not shown). Clearly, there is a threshold of repression, mediated by a minimal number of functional *SEs* that is required to restore viability by repressing *brk* transcription sufficiently. Judged by the combinations tested in this study, we found expression patterns that very closely resembled the wild type *brk* expression pattern in the case of the constructs featuring wild type *SE3&4&8*, *SE3-6*, *SE3&4&5&7&8* or *SE3-8*. These constructs rescued *brk* null mutant flies upon flip out of the *EGFP* cassette (data not shown).

### The Residual Medial Repression is still Dpp Signaling Mediated

**Figure 5:**
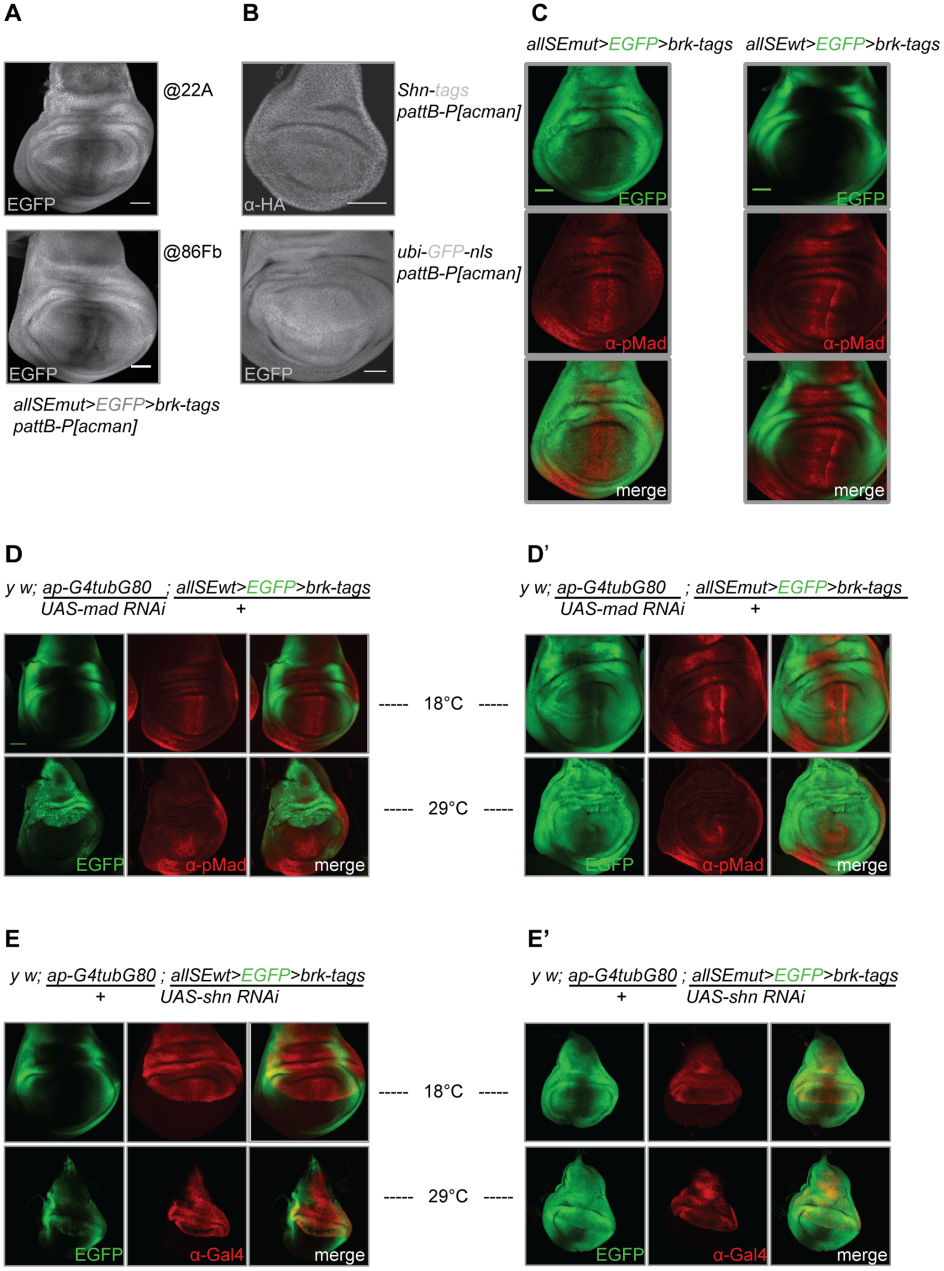
The incomplete derepression observed upon mutating all putative *SEs* still seems to be Dpp signaling dependent. (A) *EGFP* expression from the construct *allSEmut>EGFP>brk-tags* shows residual repression along the A/P compartment boundary independent of whether the construct is integrated at position 86Fb on chromosome III or at position 22A on chromosome II. (B) Ubiquitous *shn* expression from a genomic construct also cloned into the *pattB-P[acman]* integration vector (anti-HA staining) and ubiquitous *ubi-GFP-nls* expression, sequence was also cloned into the *pattB-P[acman]* integration vector. (C) *EGFP* expression patterns when expressed under the control of the endogenous *brk* regulatory region *(allSEwt>EGFP>brk-tags)* and upon mutating all potential *SEs (allSEmut>EGFP>brk-tags)*, the latter case resulting in a broadening of the Brk domain. The derepression does not take place throughout the disc. Following mutation of all the *SEs*, a slight overlap of *EGFP* expression and the anti-pMad staining can be observed. The overlap is not complete, indicating that in regions of high pMad (high Dpp signaling) there is still residual repression. (D) RNAi mediated *mad* knockdown in the dorsal compartment leads to uniform derepression of the *EGFP* readout in wing imaginal discs dissected from flies transgenic for the construct *allSEwt>EGFP>brk-tags.* The pMad staining is absent in the dorsal compartment where *mad* is knocked down via RNAi. (D’) Same as in (D), but for *allSEmut>EGFP>brk-tags.* (E) RNAi mediated *shn* knockdown in the dorsal compartment also leads to uniform derepression of the *EGFP* readout, again in wing imaginal discs dissected from flies transgenic for the construct *allSEwt>EGFP>brk-tags*. The anti-GAL4 staining marks the RNAi expression domain. (E’) Same as in (E), but for *allSEmut>EGFP>brk-tags*. Scale bars: 50 µm. *UAS-shn RNAi* and *UAS-mad RNAi*: pictures taken with identical magnification.

We had noted that even upon mutating all 13 *SEs* in the *brk* locus, medial repression of *brk* remained (Fig. 2J, L). Neither the landing site where the construct was integrated (Fig. 5A), nor elements within the *pattB-P[acman]* integration vector (Fig. 5B) seem to be the source for the residual repression.

Combining the EGFP fluorescence with a staining against pMad, a marker for Dpp pathway activity, revealed that *EGFP* expression and high levels of Dpp signaling were mutually exclusive (Fig. 5C). RNAi mediated knockdown of both Dpp pathway mediators *mad* and *shn* led to uniform *EGFP* expression in the compartment where the RNAi was active (Fig. 5D, E). These results suggest that the residual medial repression is still mediated by Dpp signaling.

**Figure 6:**
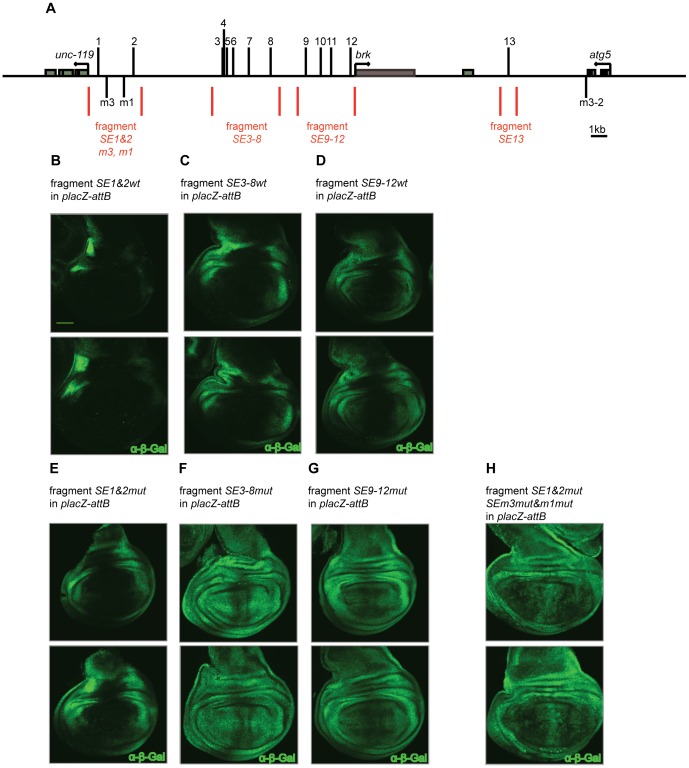
Mutating *SEs* that show an even more degenerate consensus increases *brk* derepression. (A) Schematic overview of the *brk* locus. The four fragments covering the 13 *SEs*, as well as the additionally identified *SEs*, which show a single bp substitution if compared to the perfect consensus are indicated ([2], [8]; *SEm3*, *SEm1* and *SEm3-2)*. (B)-(D) Genomic fragments covering the wild type *SE* combinations *SE1&2*, *SE3-8*, *SE9-12*. (E)-(G) Similar fragments as shown in (B)-(D) after mutating the *SEs*. (H) The genomic fragment featuring mutated *SE1&2* as well as additional mutations in *SEm3* and *SEm1*. The fragment featuring *SE13* is not shown, as no expression could be observed with *SE13mut*. Anti-β-Gal stainings were performed. Scale bar: 50 µm. Pictures were taken with constant confocal settings.

There are several possible mechanisms. It could be that the regulation occurs via a miRNA that targets the *brk* mRNA. In the BAC based constructs, sequences of the 5′ and 3′ UTR are present. To test this, we used a simplified assay, comprising genomic fragments containing either wild type or mutated *SEs* (*SE1&2wt/mut*, *SE3-8wt/mut*, *SE9-12wt/mut* and *SE13mut*) in combination with a *lacZ* reporter; *placZ-attB*; Fig. 6A). In these fragments the regulation of *lacZ* should be independent of any miRNA. The expression driven by the wild-type fragments was reminiscent of the endogenous *brk* expression (Fig. 6B–D). The exact expression varied, which is entirely consistent with previous finding that the net balance of *SE* and enhancer activities determines the *brk* expression levels [14]. Similar to the BAC results (Fig. 2J), when the *SEs* were mutated the expression domain expanded medially, however, some repression remained (Fig. 6E–G). This data contradicts a putative miRNA mediated posttranscriptional regulation.

### *SEs* with a more Degenerate Consensus might be Biologically Relevant

In the case of the fragment comprising *SE1&2mut*, the effect on the gradient upon mutating the *SEs* was particularly weak when compared to the wild type expression pattern observed with functional *SE1&2* (Fig. 6B, E). The residual repression is not due to incomplete inactivation of the *SE1&2* in this fragment, as the mutated sites no longer support pMad-Med-Shn complex formation (Fig. S2 A). As the *SE1&2mut* fragment displayed the highest levels of incomplete derepression when compared to other fragments lacking functional *SEs,* we decided to focus on this fragment for further experiments. We reasoned that a likely explanation for the incomplete derepression was the presence of additional sites that were not detected, even with our more relaxed consensus sequence (compare Fig. 1C to [2], [8]). Such more degenerate *SEs* (low affinity binding sites) probably only get bound in regions of highest Dpp signaling activity (i.e. medially), explaining the restricted occurrence of the residual repression to the A/P compartment boundary. Starting from the original consensus [2], [8] and systematically allowing point mutations at each position of the 16 bp, we found two *SEs* in the genomic fragment with *SE1&2* that each show a single mismatch at position one or three, the revised consensus sequence is either NRCGNC (N)_5_ GTCTG (*SEm1*) or GRNGNC (N)_5_ GTCTG (*SEm3*). When tested in our EMSA, these motifs indeed showed low but clearly detectable levels of pMad-Med-Shn complex formation (Fig. S2 B). To validate the sites *in vivo*, we mutated these degenerate *SEs* in the context of the fragment *SE1&2mut*. Expression of *lacZ* became more uniform, and medial repression was effectively abrogated (Fig. 6H). However, since in fragments *SE3-8mut* and *SE9-12mut* there is also some residual medial repression (Fig. 6F, G) we do not think that *SEm1* and *SEm3* explain the repression seen in the BAC construct *allSEmut>EGFP>brk-FLAG-HA-strepII*.

Consistent with findings that identify a different *SE* variant to be active in Dpp-dependent repression [15], [19] our results show that the current *SE* consensus has been defined too strictly. More degenerate *SEs* may be relevant *in vivo* in the regions of high signaling activity and hence might serve as important determinants of *brk* expression.

## Discussion

### A Precise and Robust Technique to Quantify the Contribution of Individual *brk SEs* to Dpp Mediated Repression

To understand the role of the *brinker silencer elements* in growth and patterning of the wing we manipulated them in a genomic context, thereby changing the *brinker* gene’s sensitivity to Dpp signaling on the transcriptional level.

Making use of genomic constructs featuring between zero and 13 functional consensus *SEs* in their endogenous context as well as a differently labeled internal control as a reference, we developed a quantification method that allowed us to very precisely quantify the effect of single *SEs* or *SE* combinations on the Brk gradient.

Consistent with the idea that the *SEs* seem incapable of exerting repression over long distances [15] we found that in a genomic context, a single functional *SE* is not sufficient to reproduce the endogenous *brk* expression pattern. In other words, a single functional *SE* is not able to repress the enhancers that are dispersed over the *brk* locus. Our results indicate that the presence of functional *SE3&4* has a significantly stronger effect on target gene repression than the presence of functional *SE1&2*. Furthermore, *SE10* has a stronger effect than the combination of *SE1&2*. Along the same lines, *SE4* causes a more potent repression than *SE10*. *SE4* is also stronger than *SE3*, as the presence of *SE4* alone causes more or less similar repression as the combination of *SE3&4*. *SE3* might be exceptionally weak and therefore does not detectably add to the effect of *SE4*.

The effect of the different *SEs* on *brk* repression may be influenced by a combination of factors, including the strength of individual *SEs* and the strength of the enhancers. Both the proximity of the *SEs* to the enhancers as well as the proximity of the *SEs* and enhancers to *brk’s* transcriptional start may play a role. In the future, it will be interesting to analyze more in detail the enhancers in the *brk* locus. Furthermore, it is conceivable that individual enhancer-*SE* combinations may show some tissue specific responsiveness to Dpp signaling. *SE13,* which does not seem to contribute to *brk* repression in the wing imaginal disc, may play a role in other tissues (G. Pyrowolakis, unpublished).

In comparison to *Drosophila melanogaster*, which features a relatively high number of *SEs*, other insect species contain significantly less such regulatory elements in the *brk* locus [20]. This interesting evolutionary observation might offer nice models for future investigations of the architecture of *SEs* and enhancers on a simpler level.

### Additional, more Degenerate *SE* Motifs Seem to be Present in the *brk* Locus

Mutating all the consensus *SEs* in the *brk* locus results in incomplete derepression. The residual medial repression seems to be still mediated by Dpp signaling. The mechanism underlying this residual repression is still unclear, although our results suggest that it is not post-transcriptional regulation via, for example, a miRNA. Another explanation for the remaining medial repression is the existence of a novel type of Shn-dependent repressor element; or repression might also happen indirectly as a secondary effect, for example via target genes of the Dpp pathway that might repress *brk* in medial parts of the wing disc. Alternatively, residual repression could depend on the presence of additional *SEs* which might have been missed simply because the definition of the *SEs* has been too strict.

Indeed, our results indicate that the residual medial repression is (at least partly) due to the presence of *SE* motifs with a more degenerate sequence than previously anticipated, although we cannot exclude at this stage that there are additional regulatory elements of completely different nature that also contribute to *brk* regulation. Focusing on the fragment covering *SE1&2mut*, we additionally mutated two degenerate *SE* sequences. These are two of only three degenerate *SE* sequences present in the locus that showed a single bp substitution at position one or three, compared to the perfect consensus (the third one, *m3-2*, is located in an intron of *atg5*, downstream of the *brk* coding sequence; Fig. 6A and Fig. S2C–F; [2], [8]). Mutating these sites led to an expansion of the readout, proving their functionality. If we combine the relaxed consensus that we used in this study (Fig. S2D) with these additional relaxations at the two newly identified positions, we uncover 20 additional putative *SEs*: 12 *SEs* with the consensus GNNGNC (N)_5_ GNCTN and eight with the consensus NNCGNC (N)_5_ GNCTN (Fig. S2E, F) and some of these are located in the fragments covering *SE3-8* and *SE9-12*. Clearly, this consensus might be too relaxed and not all of the sites will have an effect *in vivo*. Indeed, two of these proposed novel *SEs,* which are based on a combination of the relaxed consensus with a wobble at either position one or three of the consensus, showed no complex formation when randomly picked for *in vitro* testing (Fig. S2B). However, any future dissection of the elements regulating *brk* expression will need to take potential additional sites into account.

Previous studies had used reductionist approaches to elucidate the mechanisms underpinning *brk* expression. Taking advantage of technological advances, we analyzed the role of the *SEs* in the context of the entire locus rather than in an isolated fragment. While on the whole supporting the existing model, our results indicate that it is necessary to revise the notion of the *SE* as a motif with a strict consensus. The obvious explanation for our observations would be that in regions of high signaling, components of the repression complex (pMad-Medea-Shn) bind to sites that do not have the optimal sequence, although it remains to be shown that other regions of the *brk* regulatory region also contain such degenerate *SEs* (obviously, ChIP with Shn would be an experiment to test this, although it is not a straightforward approach and beyond the scope of this work). It may even be a general biological phenomenon that sites diverging from the perfect consensus are bound by the pathway mediators in regions of maximal signaling activity and that this will affect the expression of target genes. This has implications for the interpretation of large scale CHIP-on-CHIP and CHIP-seq datasets and serves as a note of caution against assuming that a consensus sequence can have no wobble.

## Materials and Methods

### Electrophoretic Mobility Shift Assays (EMSA)

Production of protein extracts, labeling of DNA and EMSAs were performed as described in [8] with minor modifications. Briefly, *Drosophila* S2 cells were transfected with combinations of plasmids encoding *mad*, *medea*, *tkv QD* (a constitutive active version of the type I Dpp receptor Tkv) and *shnCT* (a fragment comprising the 637 C-terminal amino acids of Shn). Cells were harvested three days post transfection and lysed in in 100 mM Tris (pH 7.8), 1 mM DTT, and 0.5% TritonX100 supplemented with a protease inhibitor cocktail (Complete, Roche) for 10 min at 4°C and cleared extracts were directly subjected to DNA binding assays. DNA probes were generated by annealing and filling in partially overlapping 24 nt-long oligonucleotides in the presence of [a-32P]dATP. Binding reactions were performed in 25 µl of 100 mM KCl, 20mM HEPES (pH 7.9), 20% glycerol, 1 mM DTT, 0.3% BSA, 0.01% NP40 containing 10,000 cpm probe, 1 µg dIdC and cleared S2-protein extracts. After incubation for 40 min at 4°C, reactions were analyzed by nondenaturing 4% polyacrylamide gel electrophoresis followed by autoradiography.

### Cloning and BAC Recombineering

Genomic fragments comprising the *SEs* were PCR amplified from *y w* genomic DNA. The original consensus sequence GRCGNC (N)_5_ GTCTG was expanded to GNCGNC (N)_5_ GNCTN. The QuikChange Site-Directed Mutagenesis Kit (Stratagene) was used for the introduction of 5 bp substitutions, resulting in the sequence GNatNC (N)_5_ tNagN. The *EGFP* stop cassette was derived from the plasmid *pEGFP-vasaφC31* [17], including an SV40 trailer and an *hsp70* 3′UTR. The cassette is flanked by one wild type and one shortened *FRT* site, the latter consisting of a single *FRT* repeat. As tags, the combinations 3x V5-, 6x His- (wild type *mCherry* construct) and 2x FLAG-, 3x HA-tags, strep-tag II (for all the other constructs) were used. The BAC covering the *brk* and *shn* loci were ordered from BACPAC Resources and BAC isolation was done following the protocol provided.

The two step BAC recombineering strategy, based on galK positive/negative selection, was performed as published [21]. Depending on the size of the modifications to be introduced and the genomic surroundings of the targeted regions (e.g. repetitive sequences), homology arms ranging from 50 bp to 2.72 kb were used.

Critical construct features were sequenced.

Primers are available on request.

### BAC Transgenesis for *Drosophila*

The BAC sequence of interest was transferred into the integration vector *pattB-P[acman]* [16], comprising 500 bp homology arms for the retrieval of the corresponding loci, linearized by BamHI restriction digest. For high yield DNA amplification prior to injection into *Drosophila* embryos, the constructs were transformed into TransforMaxTM Epi300TM electrocompetent cells (EPICENTRE). For injection, the BAC DNA was purified using the QIAGEN Large Construct Kit.

The landing sites 86Fb (chromosome 3R) and 22A (chromosome 2L) were used for ΦC31 integrase mediated site-specific integration [17].

### Immunohistochemistry in Wing Imaginal Discs

Wing imaginal discs dissected from crawling third instar larvae were fixed for 25′ in 2% FA at RT on a rotor. After washing, they were incubated with the primary antibody O/N on a rotor at 4°C, followed by a blocking step with heat inactivated goat serum for 30′ on a rotor at RT. After addition of the secondary antibody, the discs were incubated for at least 1 hour on a rotor at RT. The discs were washed and mounted in 13.5 µl of Vectashield mounting medium (Vector Labs). When discs were used for quantification, brain discs were added as spacers and confocal pictures were taken immediately after mounting.

The following primary antibodies were used: mouse anti-β-Gal (1∶1000; Promega), DAPI (1∶100; Sigma), rabbit anti-G4 (1∶300; Santa Cruz; specificity increased by pre-incubation with disrupted third instar larvae), mouse anti-HA.11 (1∶400; Covance), rabbit anti-pMad (1∶1′000; gift from Ed Laufer, Columbia University, New York), mouse anti-Patched (1∶100; DSHB), mouse-anti-Wingless (1∶1′000; 4D4; DSHB). Secondary antibodies: Alexa Fluor antibodies (Molecular Probes).

### Data Extraction and Gradient Quantification (for a Detailed Description, see Text S1)

#### Image quality control and choice of z-sections included into the analysis

For each disc, an average z-projection was generated. A 2D masks for the choice of the sections to be included in the analysis was obtained drawing manually a Region of Interest (ROI) using ImageJ. The ROIs are always defined along the dorso-ventral compartment boundary (anti-Wingless staining) in the posterior compartment (anti-Ptc staining) of the wing disc (for an example, see Fig. 3A).

The pixel values for each z-section and for both the mCherry and the EGFP channel inside the masks were extracted and analyzed using the software Mathematica (which was also used for all the subsequent analysis). Pixel value variability between consecutive sections tended to be smaller for more central sections. We therefore developed a systematic way to quantify this variability and identify the “optimal” stack displaying a maximal stability. We finally took into account and averaged five consecutive sections around the optimal section.

For the image quality control, all discs where the Wg and Ptc domains were not clearly identifiable or no optimal section was found were discarded from the analysis.

#### Profile extraction

For the profile extraction, using again ImageJ, a 1D ROI was manually traced in the dorsal compartment, around ten pixels above the *wingless* stripe (cf. Fig. 3B). The anti-Ptc staining showed a sharp border in the posterior half of the wing disc and facilitated the identification of the center of the disc. A rigorous visual identification of the end of the pouch was, however, more complicated. We started by cutting at the intersection of the anti-Wg staining with the tissue fold which confines the pouch. The analysis of the extracted profile allowed to redefine the end of the profile by identifying the exact position of the folding (where the membrane-tagged mCherry and cytoplasmic EGFP signals decorrelated: signal abrupt increase vs. decrease, cf. Fig. 4 (a) of Text S1).

#### mCherry and EGFP channel calibration

As expected, due to a different fluorescence, the absolute values obtained for the wt constructs *allSEwt>EGFP>brk-FLAG-HA-strepII* and *allSEwt>mCherry-CAAX>brk-V5-His* were not identical. The *EGFP* readout, indeed, showed an overall higher signal than the mCherry one. A “profile calibration” step was therefore necessary to adjust the different absolute values: We defined again, in a similar way as for the optimal stack selection, a 2D mask making use of the average z projection of the five optimal sections chosen before, and we collected all the pixel fluorescence mCherry-EGFP pairs for 35 wild type discs (cf. Fig. 3C) resulting in a cone-shaped distribution. After data cleaning and fitting (cf. Fig. 3D), we obtained the calibration profile by computing a linear fit of the data. We note that the values exceeding the threshold value of 0.27 in the mCherry channel were cut because of a decorrelation of the signal.

### RNAi Mediated Knockdown of Shn and Mad

The *apG4* driver was used in combination with *Gal80^ts^*. RNAi against *shn*: The larvae were transferred to 29°C 48 hours AEL. RNAi against *mad*: The larvae were transferred to 29°C after a 65 hours period of egg laying at 18°C. For both experiments, the control was constantly kept at 18°C. Wandering third instar larvae were dissected.

### Rescue of brk Null Mutants

Males transgenic for the BAC constructs were crossed to *tubβ2-FLP* virgins. *Brk^XH^* virgins were then crossed to males carrying both the *brk* BAC construct and *tubβ2-FLP* (resulting in the flip out of the *EGFP* stop cassette in the male germ line). Flies were raised at 25°C. Rescued *Brk^XH^* males were scored.

### Fly Strains

The following fly strains were used in this work:

y w; sp/CyO; allSEwt>EGFP>brk-FLAG-HA-strepII

y w; allSEwt>mCherry-CAAX>brk-V5-His; MKRS/TM6B

y w; allSEwt>mCherry->brk-V5-His; MKRS/TM6B

y w; sp/CyO; SE3-8wt>EGFP>brk-FLAG-HA-strepII

y w; sp/CyO; SE34578wt>EGFP>brk-FLAG-HA-strepII

y w; sp/CyO; SE3-6wt>EGFP>brk-FLAG-HA-strepII

y w; sp/CyO; SE348wt>EGFP>brk-FLAG-HA-strepII

y w; sp/CyO; SE34wt>EGFP>brk-FLAG-HA-strepII

y w; sp/CyO; SE4wt>EGFP>brk-FLAG-HA-strepII

y w; sp/CyO; SE1&2wt>EGFP>brk-FLAG-HA-strepII

y w; sp/CyO; SE10wt>EGFP>brk-FLAG-HA-strepII

y w; sp/CyO; allSEmut>EGFP>brk-FLAG-HA-strepII

y w; allSEmut>EGFP>brk-FLAG-HA-strepII/CyO; MKRS/TM6B

y w hs-flp; sp/CyO; SE1&2wt-placZ-attB/TM6B

y w hs-flp; sp/CyO; SE1&2mut-placZ-attB/TM6B

y w; hs-flp; sp/CyO; SE3-8wt-placZ-attB/TM6B

y w hs-flp; sp/CyO; SE3-8mut-lacZ-attB/TM6B

y w hs-flp; sp/CyO; SE9-12wt-lacZ-attB/TM6B

y w hs-flp; sp/CyO; SE9-12mut-lacZ-attB/TM6B

y w hs-flp; sp/CyO; SE13mut-lacZ-attB/TM6B

y w hs-flp; sp/CyO; SE1&2&m3&m1mut-lacZ-attB/TM6B

y w hs-flp; sp/CyO; shn-FLAG-HA-BIO-pattB-P[acman]/TM6B

yw hs-flp; sp/CyO; ubi-GFP-nls-pattB-P[acman]

y w hs-flp; sp/CyO; tubβ2-flp/TM6B

y w hs-flp; apG4 tubG80ts/Cyo; MKRS/TM6B

*y w; UAS-madRNAi/CyO;+*(VDRC transformant-ID Nr. 10970)

*y w; +; UAS-shnRNAi/TM6B* (VDRC transformant-ID Nr. 3226)

Brk^XH^; +; +.

## Supporting Information

Figure S1 **Silencing complex formation can be observed on all 13** ***SEs.*** EMSA performed for the 13 predicted *SEs* in the *brk* locus. Analogously to Fig. 1D binding of each labelled DNA was tested in three reactions/lanes. Lane 1: control; mock transfected cells. Lane 2: Smad complex formation; extracts containing TkvQD, Mad and Medea (TMM). Lane 3: Silencing complex formation; extracts containing TMM and ShnCT (S). Open arrow: Mad-Med shift, closed arrow: Mad-Med-ShnCT super shift.
(TIF)

Figure S2 **The residual repression observed in the case of the fragment covering** ***SE1&2*** **is not due to incomplete inactivation of these** ***SEs*** **, but rather due to additional, more degenerate** ***SEs*** **present in this region.** (A) While the wild type *SE1* and *SE2* are bound by the silencing complex, complex formation is clearly abolished upon mutating these *SEs*. Lane 1: control; mock transfected cells. Lane 2: Silencing complex formation; extracts containing TMM and ShnCT (S). Open arrow: Mad-Med shift, closed arrow: Mad-Med-ShnCT super shift. (B) *SEm1* and *SEm3* show silencing complex formation *in vitro*, while two additional potential *SEs* that are even more degenerate in their consensus (combination of our relaxed consensus and additionally allowing for a mismatch at position 1; termed *SEm1a* and *SEm1b*) show no complex formation, indicating that the consensus can only be relaxed so far and still allow complex formation. *SEm1a* and *SEm1b* are indicated in (F). Lane 1: control; mock transfected cells. Lane 2: Smad complex formation; extracts containing Mad, Medea (MM) and TkvQD (T). Lane 3: Silencing complex formation; extracts containing TMM and ShnCT (S). Open arrow: Mad-Med shift, closed arrow: Mad-Med-ShnCT super shift. (C) The ten *SEs* elements identified with the original consensus sequence [2], [8]. (D) The more relaxed *SE* consensus used for this study results in three more *SEs* in the *brk* locus (*SE9*, *SE11* and *SE12*). (E) Allowing a mismatch at position three, again combined with our relaxed consensus shown in (D) results in 12 additional, potential *SEs*, including *SEm3* (Fig. 6A, H).(F) Allowing a mismatch at position one, combined with our relaxed consensus shown in (D) results in eight additional, potential *SEs*, including *SEm1* (Fig. 6A, H).
(TIF)

Text S1 **Brk images quantification and data analysis.**This supporting text provides a detailed description of how the analysis of the Brk gradients was performed, including the systematic definition of a 1D ROI, the calibration of the profiles and the quantification of the profiles.
pdf
