# Supplementary figures and images for: Manipulating the Sensitivity of Signal-Induced Repression: Quantification and Consequences of Altered Brinker Gradients
Source: PLoS One. 2013 Aug 8;8(8):e71224. doi: 10.1371/journal.pone.0071224 (PMC3738585; doi:10.1371/journal.pone.0071224)

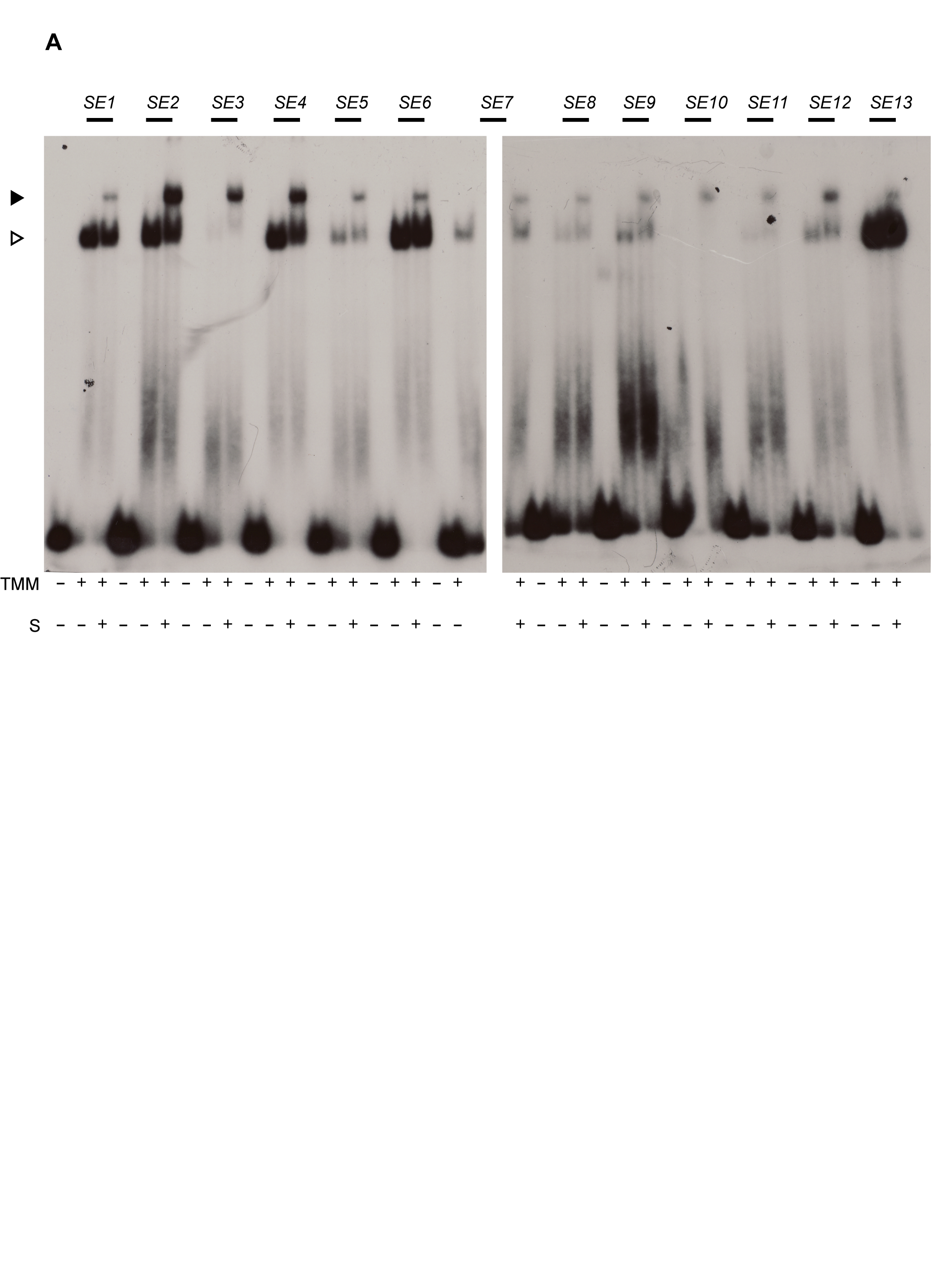

Supplement: Figure S1 — Silencing complex formation can be observed on all 13 SEs. EMSA performed for the 13 predicted SEs in the brk locus. Analogously to Fig. 1D binding of each labelled DNA was tested in three reactions/lanes. Lane 1: control; mock transfected cells. Lane 2: Smad complex formation; extracts containing TkvQD, Mad and Medea (TMM). Lane 3: Silencing complex formation; extracts containing TMM and ShnCT (S). Open arrow: Mad-Med shift, closed arrow: Mad-Med-ShnCT super shift. (TIF) [file pone.0071224.s001.tif]

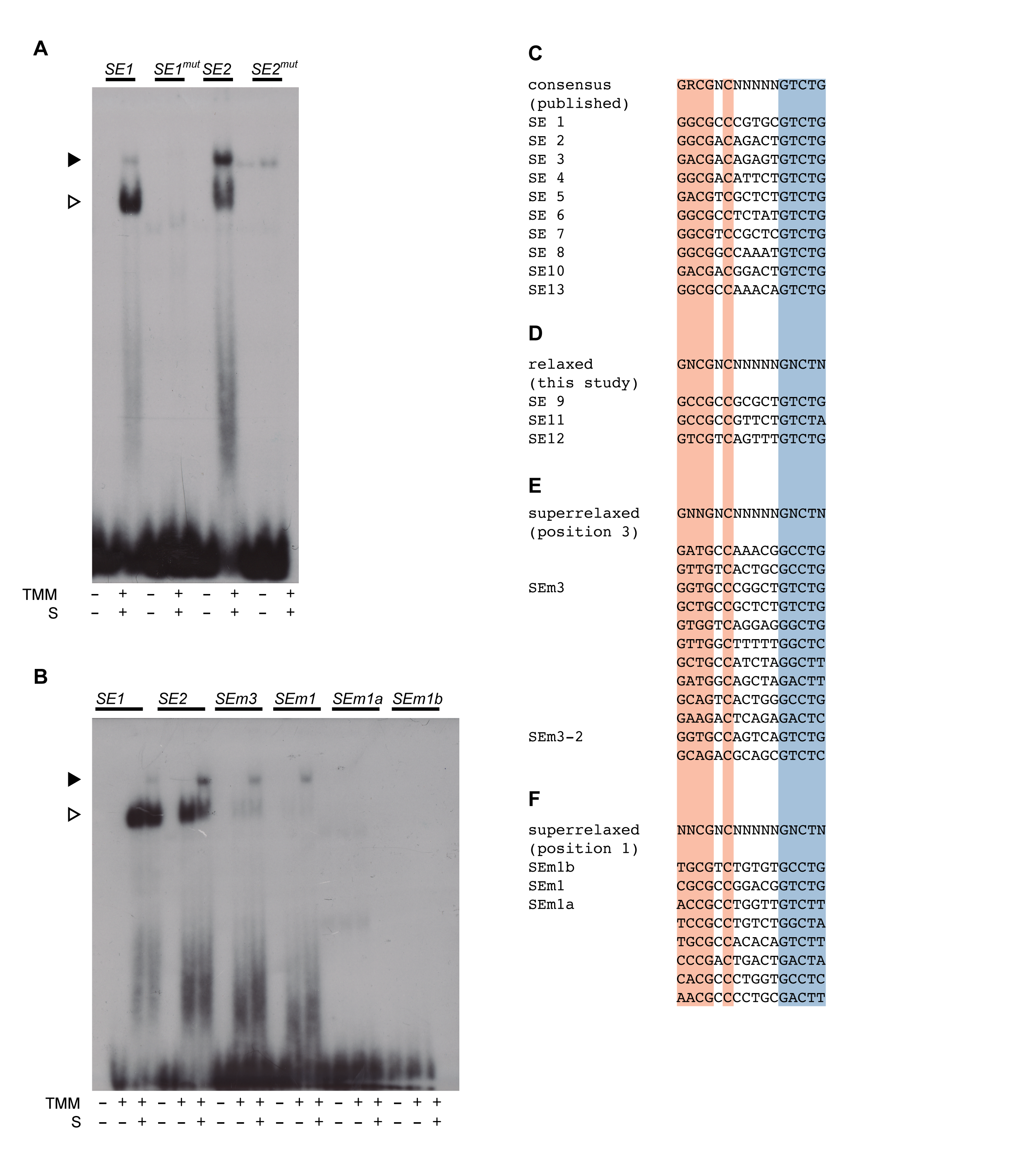

Supplement: Figure S2 — The residual repression observed in the case of the fragment covering SE1&2 is not due to incomplete inactivation of these SEs , but rather due to additional, more degenerate SEs present in this region. (A) While the wild type SE1 and SE2 are bound by the silencing complex, complex formation is clearly abolished upon mutating these SEs. Lane 1: control; mock transfected cells. Lane 2: Silencing complex formation; extracts containing TMM and ShnCT (S). Open arrow: Mad-Med shift, closed arrow: Mad-Med-ShnCT super shift. (B) SEm1 and SEm3 show silencing complex formation in vitro, while two additional potential SEs that are even more degenerate in their consensus (combination of our relaxed consensus and additionally allowing for a mismatch at position 1; termed SEm1a and SEm1b) show no complex formation, indicating that the consensus can only be relaxed so far and still allow complex formation. SEm1a and SEm1b are indicated in (F). Lane 1: control; mock transfected cells. Lane 2: Smad complex formation; extracts containing Mad, Medea (MM) and TkvQD (T). Lane 3: Silencing complex formation; extracts containing TMM and ShnCT (S). Open arrow: Mad-Med shift, closed arrow: Mad-Med-ShnCT super shift. (C) The ten SEs elements identified with the original consensus sequence [2], [8]. (D) The more relaxed SE consensus used for this study results in three more SEs in the brk locus (SE9, SE11 and SE12). (E) Allowing a mismatch at position three, again combined with our relaxed consensus shown in (D) results in 12 additional, potential SEs, including SEm3 (Fig. 6A, H).(F) Allowing a mismatch at position one, combined with our relaxed consensus shown in (D) results in eight additional, potential SEs, including SEm1 (Fig. 6A, H). (TIF) [file pone.0071224.s002.tif]
